# Treatment of comorbid anxiety symptoms and insomnia in patients with schizophrenia: A review of Pros and Cons.

**DOI:** 10.1192/j.eurpsy.2023.595

**Published:** 2023-07-19

**Authors:** E. Román, A. González-Rodríguez, M. Natividad, E. Izquierdo, A. Guàrdia, E. Calvo, J. A. Monreal

**Affiliations:** ^1^Mental Health, Mutua Terrassa University Hospital. Fundació Docència i Recerca Mutua Terrassa. University of Barcelona (UB); ^2^Mental Health, Mutua Terrassa University Hospital. Fundació Docència i Recerca Mutua Terrassa. University of Barcelona (UB). CIBERSAM; ^3^Mental Health, Mutua Terrassa University Hospital Fundació Docència i Recerca Mutua Terrassa. University of Barcelona (UB); ^4^Mental Health, Mutua Terrassa University Hospital. Fundació Docència i Recerca Mutua Terrassa. University of Barcelona (UB). CIBERSAM. Institut de Neurociències. UAB, Terrassa, Spain

## Abstract

**Introduction:**

Recent studies report a prevalence of comorbid anxiety disorder of 38% and 80% of sleep disorders in schizophrenia. Insomnia has been associated with worsening of psychopathological symptoms and increased hospitalization rates.

**Objectives:**

The aim of our work was to review mechanisms of action and the use of benzodiazepines, GABAergic drugs and melatonin for the treatment of anxiety and insomnia in schizophrenia.

**Methods:**

We carried out a narrative review of studies focusing on the treatment of anxiety disorders and insomnia in schizophrenia through PubMed and Google Scholar (2002-September 2022). The use of benzodiazepines, GABAergic drugs and melatonin in schizophrenia are discussed, illustrating them with case reports.

**Results:**

A total of 32 studies were included. (A)Benzodiazepines (BZD) work facilitating the inhibitory actions of gamma-aminobutyric acid (GABA) by binding GABA type A receptors. The beneficial effect of combined use of antipsychotics and BZD is controversial (cognitive complications, sedation, overdosing, substance use, etc). (B)GABAergic drugs: gabapentin (GP) and pregabalin (PG) (structurally related molecules), have no direct GABAergic action and act inhibiting voltage-gated calcium channels. The efficacy of GP and PG in the treatment of anxiety symptoms in schizophrenia is understudied. Positive effects of GP in schizophrenia suffering from restless legs syndrome receiving clozapine. (C )Melatonin, an hormone produced in the pineal gland has been used to treat insomnia. Positive effects on metabolic syndrome and cardiovascular risk factors have been reported. Several works considered it an alternative in schizophrenia.

**Conclusions:**

Few evidence is available on the use of BZD, GP, and PG in schizophrenia. Melatonin is a promising compound to treat insomnia.

**Disclosure of Interest:**

None Declared

